# Curcumin Attenuates Bisphenol F-Induced Osteoporosis and Osteogenic Dysfunction via PI3K/AKT Pathway Activation

**DOI:** 10.3390/nu18142335

**Published:** 2026-07-16

**Authors:** Yue Su, Hao Li, Keyi Zhang, Na Liang, Xiongfei Hu, Gang Luo, Xiuhong Yang, Qiang Wang, Lin Xiao

**Affiliations:** 1 Xiangya School of Public Health, Central South University, Changsha 410078, China; 246911035@csu.edu.cn (Y.S.); 236912060@csu.edu.cn (H.L.); zhangky@csu.edu.cn (K.Z.); 236912047@csu.edu.cn (N.L.); luogang@csu.edu.cn (G.L.); 2 Hunan Provincial Center for Disease Control and Prevention (Hunan Academy of Preventive Medicine), Changsha 410153, China; 3 Hunan Prevention and Treatment Institute of Occupational Diseases, Changsha 410007, China; hxf20090801@163.com (X.H.); yxh502@sina.com (X.Y.)

**Keywords:** osteoporosis, bisphenol F, curcumin, endocrine-disrupting chemical, PI3K/AKT pathway

## Abstract

**Background**: Bisphenol F (BPF), a widely used bisphenol A substitute and emerging endocrine disruptor, has been implicated in bone loss; however, its underlying mechanisms remain unclear. Curcumin (CUR) has shown beneficial effects on bone metabolism, but its protective role against BPF-induced osteogenic impairment requires further validation. This study investigated the involvement of the PI3K/AKT signaling pathway in BPF-induced osteoporosis and osteogenic dysfunction, and whether CUR could alleviate these effects through modulation of this pathway. **Methods**: Network toxicology, network pharmacology, and molecular docking were first used to predict potential targets and signaling pathways. A rat model of BPF-induced osteoporosis and a BPF-treated MC3T3-E1 osteogenic impairment model were then established to evaluate the effects of CUR. Bone mineral density, trabecular microarchitecture, serum bone metabolism markers, and osteogenic protein expression were assessed in vivo. In vitro, alkaline phosphatase activity, mineralization, and key signaling proteins were measured. The PI3K/AKT inhibitor LY294002 was used to verify pathway involvement. **Results**: Computational analyses suggested that BPF and CUR were closely associated with the PI3K/AKT pathway. Experimentally, BPF reduced bone mass, disrupted trabecular structure, and suppressed osteogenic differentiation, accompanied by downregulation of osteogenic markers and PI3K/AKT signaling. Notably, BPF reduced femoral BMD by 10.8%, whereas CUR restored approximately 43.5% of the BPF-induced loss. CUR significantly reversed these effects, whereas LY294002 abolished the protective actions of CUR. **Conclusions**: BPF may impair bone formation and promote osteoporosis by suppressing PI3K/AKT signaling, while CUR alleviates these effects through pathway activation. These findings provide mechanistic evidence supporting CUR as a potential intervention against BPF-related bone toxicity.

## 1. Introduction

Osteoporosis (OP) is a systemic skeletal disorder characterized by low bone mass and deterioration of bone microarchitecture, resulting in increased bone fragility and fracture risk [1]. With population aging and increased life expectancy, OP has become a major global public health concern, affecting approximately 200 million people worldwide [2]. Because OP is often asymptomatic in its early stages, it is frequently diagnosed only after a fracture occurs, thereby imposing substantial physical, psychological, and socioeconomic burdens on patients and healthcare systems [3].

Bone homeostasis depends on the dynamic balance between bone formation by osteoblasts and bone resorption by osteoclasts [3]. Disruption of this balance contributes to the pathogenesis of OP, which is influenced by multiple factors, including estrogen deficiency, oxidative stress, inflammation, cellular senescence, and epigenetic dysregulation [4]. In recent years, increasing attention has been paid to environmental determinants of bone health, particularly exposure to endocrine-disrupting chemicals (EDCs), which may interfere with skeletal development and remodeling [5].

Bisphenol F (BPF) has emerged as a major substitute for bisphenol A (BPA), a well-recognized endocrine disruptor with established skeletal toxicity [6]. BPF is widely used in the manufacture of epoxy resins and coatings and is commonly detected in consumer products and human biological samples [7,8]. For example, BPF has been detected in 66.5% of urine samples from adults and children in the United States [9], and in 74.6% of adult urine samples from South China [10]. The widespread use of BPF reflects the trend of replacing BPA with structurally similar analogs that are often introduced without comprehensive toxicological evaluation [7]. Accumulating evidence suggests that BPF exhibits endocrine-disrupting properties comparable to those of BPA and is associated with cardiovascular disease [11], lipid metabolism disorders [12], and reproductive toxicity [13]. More importantly, recent studies have linked BPF exposure to reduced bone mineral density in children and impaired osteoblast differentiation [14,15]. However, the molecular mechanisms through which BPF disrupts osteogenesis remain insufficiently understood.

Osteoporosis is now recognized as a multifactorial disease involving not only altered bone remodeling but also oxidative stress, inflammation, and metabolic dysregulation [16,17,18]. These processes can promote osteoclastogenesis and suppress osteoblast differentiation, thereby accelerating bone loss [19]. In addition, intracellular signaling pathways that regulate cell survival and differentiation, such as PI3K/AKT, play pivotal roles in osteogenesis and skeletal maintenance [20]. Whether BPF affects bone metabolism through this signaling axis remains unclear and warrants further investigation.

Curcumin (CUR), a bioactive polyphenol derived from Curcuma longa, has attracted considerable attention because of its anti-inflammatory, antioxidant, anticancer, and anti-aging properties [21,22]. Beyond its broad biological activities, CUR has been reported to exert protective effects in OP by promoting osteogenic differentiation, enhancing angiogenesis, and modulating bone remodeling through inhibition of osteoclast activity and stimulation of osteoblast function [23,24]. Nevertheless, its ability to counteract BPF-induced osteogenic impairment, and the underlying molecular mechanisms involved, have not been fully elucidated.

In the present study, we integrated network toxicology, network pharmacology, and molecular docking to predict the potential targets and pathways involved in BPF-induced skeletal toxicity and CUR-mediated protection. We then established a rat model of BPF-induced osteoporosis and an MC3T3-E1 osteogenic differentiation impairment model to validate the predictive findings in vivo and in vitro. Furthermore, the PI3K/AKT inhibitor LY294002 was used to verify whether this signaling pathway mediates the protective effects of CUR against BPF-induced osteogenic dysfunction.

## 2. Materials and Methods

### 2.1. Chemicals and Reagents

Dimethyl sulfoxide (DMSO) and α-minimum essential medium (α-MEM) were purchased from Adamas Reagent Co., Ltd. (Shanghai, China). Fetal bovine serum (FBS) was obtained from SenBeiJia Biological Technology Co., Ltd. (Nanjing, China). Bisphenol F (BPF, ≥98%) and bisphenol A (BPA, ≥98%) were purchased from Macklin Biochemical Co., Ltd. (Shanghai, China). Curcumin (CUR, ≥97%) was obtained from Dalian Meilun Biotechnology Co., Ltd. (Dalian, China). Calcium (Ca), phosphorus (P), and alkaline phosphatase (ALP) assay kits were purchased from Nanjing Jiancheng Bioengineering Institute (Nanjing, China). The ALP staining kit and Alizarin Red S (ARS) staining kit were obtained from Beyotime Biotechnology (Shanghai, China) and OriCell (Cyagen, Guangzhou, China), respectively. β-glycerophosphate, dexamethasone, ascorbic acid, and LY294002 were purchased from GLPBIO (Montclair, CA, USA).

### 2.2. Animals and Treatment

Male Sprague-Dawley rats (6–8 weeks old, weighing 200 ± 20 g, certificate No. SCXK (Xiang) 2024-0021) were purchased from Hunan Tianqin Biotechnology Co., Ltd. (Changsha, China) and housed under specific pathogen-free conditions at 25 ± 2 °C, 50 ± 5% humidity, with a 12 h light/dark cycle. All experimental procedures were approved by the Animal Welfare and Ethics Committee of the Hunan Occupational Disease Prevention and Treatment Institute (HNZFY-FE-0303).

After one week of acclimatization, the rats were randomly assigned to five groups (*n* = 8 per group) for the first-phase (dose–response) experiment. This sample size was determined based on previously published studies using comparable rat models of skeletal toxicity and osteometabolic injury [25,26], as well as standard toxicological experimental design principles. The experiment comprised a 4-week gavage treatment: control group (vehicle, 1% DMSO), low-dose BPF group (LBPF, 5 mg/kg/day), medium-dose BPF group (MBPF, 10 mg/kg/day), high-dose BPF group (HBPF, 20 mg/kg/day), and BPA group (20 mg/kg/day BPA, positive control). The BPF doses were selected based on a previous 28-day oral toxicity study in rats [27], in which 5 mg/kg BPF significantly reduced bone elasticity, together with the reported lowest observed adverse effect level (LOAEL) of 20 mg/kg. BPA was included as a positive control because it is the most extensively studied bisphenol analogue with well-documented adverse effects on bone metabolism. The primary objective of the first phase was to characterize the dose-dependent osteotoxic effects of BPF and to identify the optimal exposure concentration for the subsequent intervention study. At the end of the first phase, all rats were fasted for 12 h, anesthetized with 2% sodium pentobarbital (40 mg/kg, i.p.; Shanghai Yuanye Bio-Technology Co., Ltd., Shanghai, China), and euthanized. Blood serum and femoral tissues were collected for biochemical assays, bone mineral density (BMD) measurement, histopathological examination, and protein expression analysis.

Based on the dose–response findings from the first phase (see Section 3), the HBPF dose (20 mg/kg) was selected as the optimal osteotoxic challenge for the second-phase intervention experiment. A new cohort of rats was randomly assigned into three groups (*n* = 8 per group) and subjected to an 8-week gavage treatment: control group (vehicle, 1% DMSO), BPF group (20 mg/kg/day BPF), and BPF + CUR group (20 mg/kg/day BPF plus 100 mg/kg/day CUR). The dose of CUR was selected based on a previous study demonstrating that 100 mg/kg CUR significantly ameliorated glucocorticoid-induced osteoporosis in rats after 60 days of oral administration [28]. Body weight was monitored weekly throughout the experiment, and all animals had free access to food and water. At the end of the second phase, rats were fasted for 12 h, anesthetized, and euthanized following the same protocol described above. Serum and femoral tissues were collected for biochemical assays, bone mineral density measurement, histopathological examination, and protein expression analysis.

### 2.3. Blood Biochemical Indicators

Arterial blood was collected and centrifuged at 1200× *g* for 10 min at 4 °C to obtain serum. Serum Ca, P, and ALP levels were measured using commercial assay kits (Nanjing Jiancheng Bioengineering Institute, Nanjing, China) according to the manufacturers’ instructions.

### 2.4. Measurement of Bone Mineral Density (BMD)

Dual-energy X-ray absorptiometry (DXA) was performed on anesthetized rats. The quantification of BMD was performed using Vision DXA (GE Lunar Prodigy, Madison, WI, USA) with a manually drawn region of interest (ROI) on the left femur. BMD measurement was performed according to the manufacturer’s protocol.

### 2.5. Hematoxylin and Eosin (HE) Staining

Femoral bones were fixed in 4% paraformaldehyde (Servicebio, Wuhan, China), decalcified in 10% ethylenediaminetetraacetic acid (EDTA; Servicebio, Wuhan, China), dehydrated in graded ethanol, and embedded in paraffin. Longitudinal sections (5 μm thick) were cut and mounted on poly-L-lysine-coated slides, followed by hematoxylin and eosin (Servicebio, Wuhan, China) staining to evaluate histopathological changes. All staining procedures were performed according to standard histological protocols.

### 2.6. Cell Culture

MC3T3-E1 cells were obtained from the Cell Bank of the Chinese Academy of Sciences (Kunming, China) and cultured in α-MEM supplemented with 10% FBS and 100 U/mL penicillin/streptomycin (Beyotime Biotechnology, Shanghai, China). For osteogenic induction, cells were cultured in osteogenic induction medium (OIM) consisting of basal medium supplemented with 10 mM β-glycerophosphate, 0.1 μM dexamethasone, and 50 μg/mL ascorbic acid. Cells at 60–70% confluence were used for subsequent experiments.

### 2.7. Cell Viability Assay

MC3T3-E1 cells were seeded in 96-well plates at a density of 3 × 10^3^ cells per well and cultured for 24 h to allow cell attachment. Cells were then treated with various concentrations of bisphenol F (BPF; 0, 0.001, 0.01, 0.1, 1, and 10 μM), curcumin (CUR; 0, 0.5, 1, and 2 μM), or LY294002 (0, 1, 5, 10, and 20 μM) for 48 h. The final concentration of dimethyl sulfoxide (DMSO) in all treatment groups was maintained below 0.1% (*v*/*v*). Following treatment, 10 μL of Cell Counting Kit-8 (CCK-8; APExBIO, Houston, TX, USA) solution was added to each well according to the manufacturer’s instructions, and the plates were incubated for an additional 2 h at 37 °C. The optical density (OD) was then measured at 450 nm using a microplate reader (Thermo Fisher Scientific, Waltham, MA, USA). Each concentration was tested in six technical replicates, and all experiments were independently repeated at least three times.

### 2.8. ALP Staining

MC3T3-E1 cells were seeded in 6-well plates pre-coated with 0.1% gelatin at a density of 3 × 10^4^ cells per well. After reaching approximately 80% confluence, cells were cultured in osteogenic induction medium. The experiment was conducted in two stages. In stage I, cells were divided into four groups: control, BPF (1 μM), BPF (1 μM) + CUR (2 μM), and CUR (2 μM). In stage II, cells were divided into four groups: control, BPF (1 μM), BPF (1 μM) + CUR (2 μM), and BPF (1 μM) + CUR (2 μM) + LY294002 (10 μM). Treatment concentrations were determined based on the CCK-8 assay results.

After 14 days of osteogenic induction, ALP staining was performed using a BCIP/NBT ALP staining kit (Beyotime Biotechnology, Shanghai, China) according to the manufacturer’s instructions. Briefly, cells were fixed with 4% paraformaldehyde, incubated with staining solution in the dark until color development, and observed under an inverted light microscope (Carl Zeiss, Oberkochen, Germany). The stained area was quantified using ImageJ software (version 1.54f).

### 2.9. Assessment of Cellular Mineralization

ARS staining was performed according to the manufacturer’s instructions after 21 days of osteogenic induction. MC3T3-E1 cells were seeded and treated as described in Section 2.8. After induction, cells were fixed with 4% paraformaldehyde and stained with 0.1% ARS solution for 30 min at room temperature. Mineralized nodules were visualized under a light microscope, and the mineralized area was quantified using ImageJ software. All experiments were independently repeated at least three times.

### 2.10. Western Blotting

For animal experiments, femoral tissues collected from rats were immediately frozen in liquid nitrogen and stored at −80 °C until analysis. Femoral tissues were crushed on ice using bone forceps, and bone marrow was carefully removed prior to lysis. Total protein was extracted using RIPA buffer (Applygen Technologies Inc., Beijing, China) containing protease and phosphatase inhibitors.

For cell experiments, MC3T3-E1 cells were cultured in osteogenic induction medium and treated according to the experimental grouping described in Section 2.8 for 7 days. After treatment, cells were washed twice with cold PBS and lysed using the same RIPA buffer.

The total protein concentration was determined using a BCA protein assay kit (Abbkine Scientific Co., Ltd., Wuhan, China). Equal amounts of protein were mixed with 5× SDS-PAGE loading buffer and boiled at 100 °C for 10 min, then separated by 10% SDS-PAGE and transferred onto polyvinylidene fluoride (PVDF) (Merck KGaA, Darmstadt, Germany) membranes at 300 mA for 1 h in an ice bath. After blocking with 5% non-fat milk at room temperature for 1 h, membranes were incubated overnight at 4 °C with primary antibodies against RUNX2 (1:1000; Nature Biosciences, Hangzhou, China), osteocalcin (OCN, 1:1000; ABclonal, Wuhan, China), PI3K (1:1000; ABclonal, Wuhan, China), AKT (1:1000; ABclonal, Wuhan, China), p-PI3K (1:1000; ABclonal, Wuhan, China), and p-AKT (1:1000; ABclonal, Wuhan, China). GAPDH was used as a loading control. Phosphorylated proteins (p-PI3K and p-AKT) were normalized to their corresponding total proteins (PI3K and AKT, respectively). Total proteins (RUNX2, OCN, PI3K, AKT) were normalized to GAPDH. Densitometric quantification was performed using ImageJ software (National Institutes of Health, Bethesda, MD, USA). The relative expression level of each target protein was calculated as the ratio of the target band intensity to the loading control (total protein or GAPDH) band intensity. All Western blotting procedures were performed according to standard protocols.

### 2.11. Network Toxicology Analysis

“Osteoporosis” and “osteogenic dysfunction” were used as keywords to retrieve disease-related targets from the GeneCards and OMIM databases. Targets with a relevance score ≥ 1 in GeneCards were retained. The chemical structure and SMILES information of BPF were obtained from PubChem. Potential BPF targets were retrieved from ChEMBL, CTD, SwissTargetPrediction, SEA, and TargetNet. In SwissTargetPrediction, targets with a probability > 0 were retained, and in TargetNet, targets with a probability ≥ 0.1 were included. All targets were standardized using the UniProt database, with species restricted to Homo sapiens. Duplicate entries were removed. The overlap between BPF-related and disease-related targets was identified using a Venn diagram. Overlapping targets were entered into STRING to construct a protein–protein interaction (PPI) network. The species was set to Homo sapiens, and the minimum interaction score was set to >0.7. The network was visualized using Cytoscape (version 3.10.3), and topological analysis was performed using the CytoNCA plugin. Six parameters were used: degree centrality, betweenness centrality, closeness centrality, eigenvector centrality, local average connectivity, and network centrality. Nodes with values above the median were retained after two rounds of filtering to identify hub genes. Hub gene expression was validated using the GSE35958 dataset from the GEO database by *t*-test analysis. GO and KEGG enrichment analyses were performed using DAVID (version 6.8), and the results were visualized with ggplot2 (version 4.0.0) in R (version 4.5.1).

### 2.12. Network Pharmacology Analysis

The chemical structure and SMILES information of curcumin were obtained from PubChem. Potential curcumin targets were predicted using PharmMapper (version 2017), with the species restricted to Homo sapiens. Intersecting targets between curcumin-associated targets and osteoporosis/osteogenic dysfunction-related genes were identified using a Venn diagram. These genes were imported into STRING to construct a PPI network and visualized in Cytoscape. Network modules were identified using MCODE, and hub genes were screened using CytoHubba based on the maximal clique centrality (MCC) algorithm. Core genes were defined by the overlap between the results of both algorithms. GO and KEGG enrichment analyses were performed using DAVID, and the results were visualized in R.

### 2.13. Molecular Docking

Molecular docking was used to evaluate the interactions of BPF and curcumin with the core target proteins. The three-dimensional structures of BPF and curcumin were obtained from PubChem, and crystal structures of target proteins were retrieved from the Protein Data Bank. Protein structures were preprocessed in PyMOL (version 3.1.0) by removing water molecules and adding hydrogen atoms. Docking simulations were performed using AutoDock Vina (version 1.2.7), and the conformation with the lowest binding energy was selected as the optimal binding mode. The docking results were visualized in PyMOL.

### 2.14. Statistical Analysis

All experiments were independently repeated three or more times. Quantitative data are presented as mean ± standard deviation (SD). Normality and homogeneity of variance were tested using the Shapiro–Wilk test and Levene’s test, respectively. For data meeting the assumptions of normal distribution and equal variance, comparisons among multiple groups were performed using one-way analysis of variance (ANOVA), followed by the least significant difference *t*-test (LSD-t) for post hoc pairwise comparisons when a statistically significant difference was detected. For network toxicology and network pharmacology analyses, *t*-tests were used for comparisons. Statistical analyses were performed using SPSS 25.0, GraphPad Prism 10, and R version 4.5.1. A *p* value < 0.05 was considered statistically significant.

## 3. Results

### 3.1. Network Toxicology Analysis of the Potential Mechanisms Underlying BPF-Induced Osteoporosis and Osteogenic Differentiation

**Figure 1:**
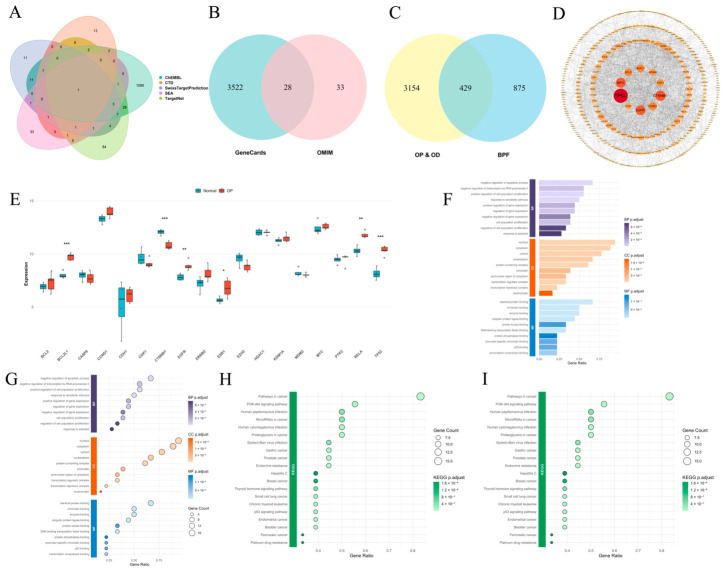
Identification of key targets and signaling pathways involved in BPF-induced osteoporosis. Target collection for BPF (**A**). Target collection for osteoporosis and osteogenic differentiation. (**B**). Venn diagram showing the intersecting targets between BPF and disease-related genes. OP: osteoporosis; OD: osteogenic differentiation (**C**). PPI network of the overlapping targets (**D**). Differential expression profiles of 18 core genes in the GSE35958 dataset. Normal: control group; OP: osteoporosis group. * *p* < 0.05, ** *p* < 0.01, *** *p* < 0.001 (**E**). Bar plot of GO enrichment analysis (**F**). Bubble plot of GO enrichment analysis (**G**). Bubble plot of KEGG pathway analysis (**H**). Bar plot of KEGG pathway analysis (**I**).

Through comprehensive screening of the ChEMBL, CTD, SwissTargetPrediction, SEA, and TargetNet databases, a total of 1304 potential molecular targets of BPF were identified. Meanwhile, disease-associated targets related to osteoporosis and osteogenic differentiation were collected from the GeneCards and OMIM databases, yielding 3583 genes. Venn diagram analysis revealed 429 overlapping genes between BPF targets and disease-related genes (Figure 1A–C), suggesting their potential involvement in BPF-mediated osteoporosis. PPI network constructed using STRING database and analyzed via Cytoscape identified the top 18 hub genes (Figure 1D). To further validate the reliability of these core targets, the GSE35958 dataset was employed to analyze their expression profiles. This dataset included 5 primary osteoporosis samples and 4 control samples. Differential expression analysis using the *t*-test demonstrated that 6 core genes (*BCL2L1, CTNNB1*, *EGFR*, *ESR1*, *RELA*, and *TP53*) showed significant differences between the two groups (*p* < 0.05). Among them, all genes except *CTNNB1* were significantly upregulated in the osteoporosis group (Figure 1E). GO and KEGG enrichment analyses of the 18 core targets were performed using the DAVID database and visualized using R software. The GO analysis suggested that these targets were primarily involved in the regulation of apoptosis, gene expression, and cell proliferation, as well as responses to xenobiotic and hormonal stimuli. These targets were mainly distributed in the nucleus and cytoplasm, particularly associated with chromatin and transcription-related complexes, and were functionally characterized by diverse protein binding activities (Figure 1F,G). KEGG pathway analysis further indicated that these targets were predominantly enriched in cancer-related pathways and key signaling pathways, notably the PI3K/AKT and p53 signaling pathways (Figure 1H,I). These findings suggest that BPF may contribute to osteoporosis by disrupting the balance between cell proliferation and apoptosis, potentially through interference with xenobiotic and hormone-related responses. Furthermore, the enrichment of the PI3K/AKT and p53 signaling pathways indicates that BPF may impair osteogenic processes and promote apoptosis of bone-related cells, ultimately leading to bone loss.

### 3.2. High-Dose BPF Induces OP in Rats

**Figure 2:**
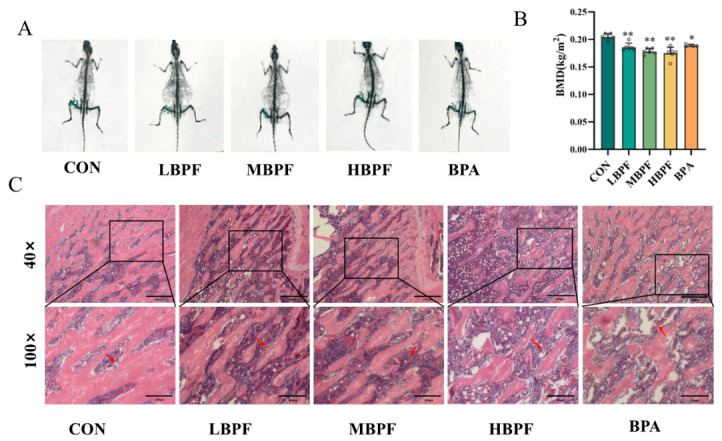
High-dose BPF induces OP in SD rats. (**A**,**B**) BMD of the proximal femur (*n* = 6). (**C**) The HE staining of the bone trabecula on the femoral neck of the proximal femur (×40 and ×100 magnification) (*n* = 3), red arrows point to deteriorated trabecular structures. Treatment groups: CON (1% DMSO vehicle control), LBPF (5 mg/kg/d BPF), MBPF (10 mg/kg/d BPF), HBPF (20 mg/kg/d BPF), BPA (20 mg/kg/d BPA). Data are presented as mean ± SD. * *p* < 0.05, ** *p* < 0.01 vs. CON.

**Figure 3:**
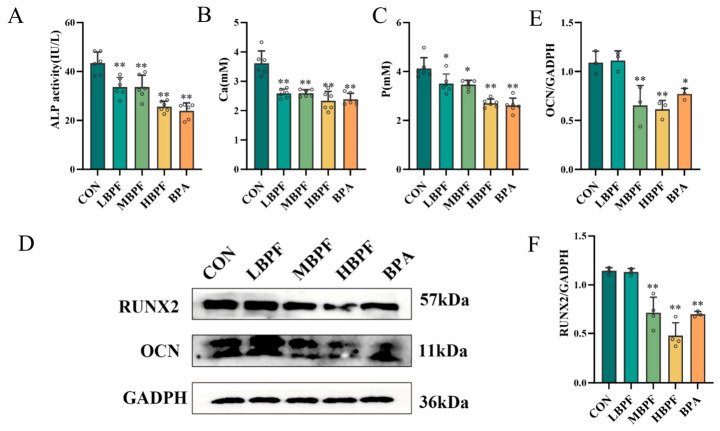
High-dose BPF induces osteoporosis by disrupting serum biomarkers and osteogenic markers in SD rats. (**A**) Serum ALP activity (IU/L) (*n* = 6). (**B**) Serum Ca levels (mM) (*n* = 6). (**C**) Serum P levels (mM) (*n* = 6). (**D**) Representative Western blotting of RUNX2, OCN, and GAPDH in femoral tissue (*n* = 3). (**E**,**F**) Quantitative analysis of RUNX2 and OCN protein levels normalized to GAPDH (*n* = 3). Treatment groups: CON (1% DMSO vehicle control), LBPF (5 mg/kg/d BPF), MBPF (10 mg/kg/d BPF), HBPF (20 mg/kg/d BPF), BPA (20 mg/kg/d BPA). Data are presented as mean ± SD. * *p* < 0.05, ** *p* < 0.01 vs. CON.

Initial experiments established that high-dose BPF administration induced significant osteoporosis in rats. As demonstrated in Figure 2, high-concentration BPF exposure markedly reduced femoral BMD, and histopathological examination via HE staining revealed thinner, disorganized, and substantially diminished trabecular bone in the HBPF group compared to controls. Concurrently, as shown in Figure 3A–C, serum levels of ALP, Ca, and P were also significantly decreased. Consistent with the osteoporotic phenotype, Western blot results indicated significant downregulation of key osteogenic marker proteins, OCN and RUNX2 (Figure 3E,F), in the HBPF group. Notably, comparative assessment with the known osteoporotic agent BPA revealed that BPF exposure caused similar detrimental effects on these skeletal parameters (BMD, serum biomarkers, bone microstructure, and osteogenic protein expression) to those induced by equivalent concentrations of BPA (Figure 2 and Figure 3). Collectively, these results demonstrate that high-concentration BPF exposure induces osteoporosis in rats.

### 3.3. Network Pharmacology Analysis of the Potential Mechanisms Underlying Curcumin-Mediated Osteoporosis and Osteogenic Differentiation

**Figure 4:**
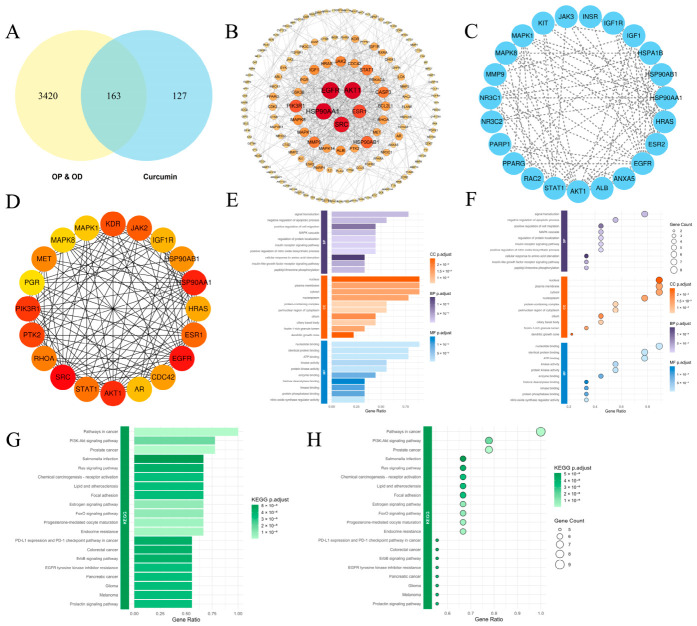
Identification of key targets and signaling pathways involved in the anti-osteoporotic effects of curcumin. Venn diagram showing the intersecting targets between curcumin and disease-related genes. OP: osteoporosis; OD: osteogenic differentiation (**A**). PPI network of the overlapping targets (**B**). Core targets identified by the MCODE algorithm (**C**). Core targets ranked by the MCC algorithm (**D**). Bar plot of GO enrichment analysis (**E**). Bubble plot of GO enrichment analysis (**F**). Bar plot of KEGG pathway analysis (**G**). Bubble plot of KEGG pathway analysis (**H**).

A total of 290 potential targets of curcumin were obtained from the PharmMapper database. By integrating the previously identified targets related to osteoporosis and osteogenic dysfunction, 163 overlapping targets between curcumin and the disease were identified (Figure 4A). These common targets were subsequently imported into the STRING database for PPI analysis. Using a minimum required interaction score > 0.7, the constructed PPI network consisted of 163 nodes and 2091 edges (Figure 4B). Hub genes within the PPI network were screened using the MCODE plugin and the CytoHubba plugin based on the MCC algorithm, with the top 20 nodes selected for analysis, and the intersection of the two methods yielded 9 core targets (Figure 4C,D). GO and KEGG enrichment analyses of these core targets were performed using the DAVID database. The GO analysis indicated that these targets were mainly enriched in signal transduction, regulation of apoptosis, cell migration, and kinase-related processes, including the MAPK cascade (Figure 4E,F). KEGG pathway analysis further demonstrated significant enrichment in cancer-related pathways and key signaling pathways, particularly the PI3K/AKT, Ras, and ErbB signaling pathways, along with pathways associated with endocrine regulation and immune response (Figure 4G,H). These findings suggest that curcumin may exert its effects by modulating signaling networks involved in cell proliferation, apoptosis, and migration, thereby potentially regulating osteogenic processes.

### 3.4. CUR Mitigates BPF-Induced OP via PI3K/AKT Pathway

**Figure 5:**
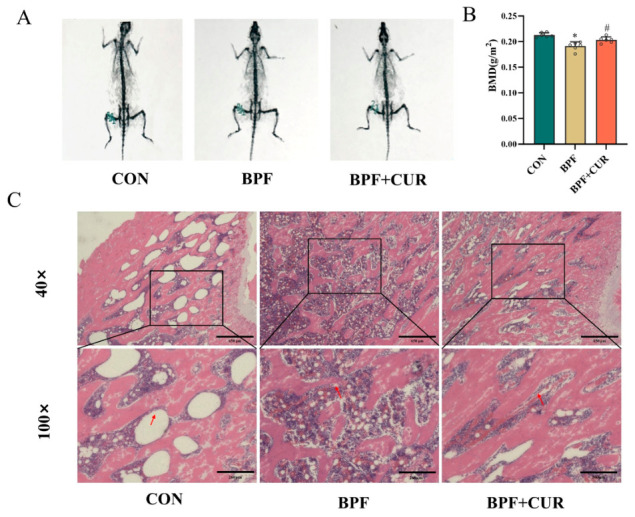
CUR ameliorates BPF-induced OP in SD rats. (**A**,**B**) BMD of the proximal femur (*n* = 6). (**C**) The HE staining of the bone trabecula on the femoral neck of the proximal femur (×40 and ×100 magnification) (*n* = 3), red arrows point to deteriorated trabecular structures. Treatment groups: CON (1% DMSO vehicle control), BPF (20 mg/kg/d), BPF + CUR (20 mg/kg/d BPF + 100 mg/kg/d CUR). Data are presented as mean ± SD. * *p* < 0.05 vs. CON; ^#^ *p* < 0.05 vs. BPF group.

**Figure 6:**
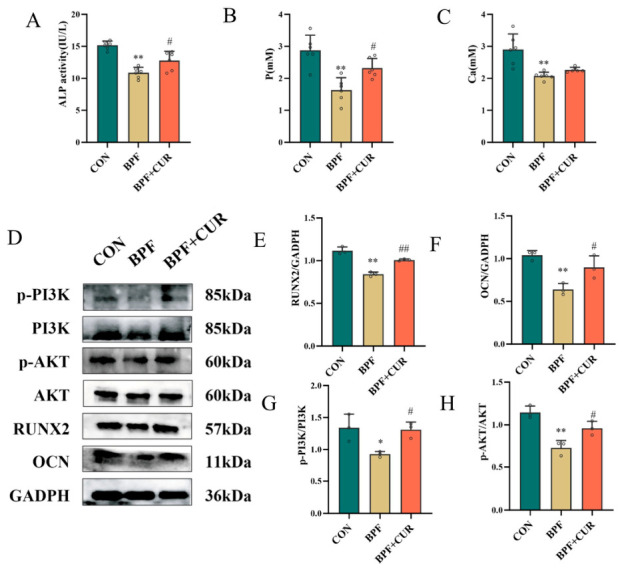
CUR ameliorates BPF-induced OP through the PI3K/AKT pathway. (**A**) Serum ALP activity (IU/L) (*n* = 6). (**B**) Serum P levels (mM) (*n* = 6). (**C**) Serum Ca levels (mM) (*n* = 6). (**D**) Representative Western blotting of p-PI3K, PI3K, p-AKT, AKT, RUNX2, OCN, and GAPDH in rat femoral tissues (*n* = 3). (**E**,**F**) Quantitative analysis of RUNX2 and OCN expression normalized to GAPDH in femoral tissue (*n* = 3). (**G**,**H**) Phosphorylation ratios of PI3K and AKT in femoral tissues (*n* = 3). Treatment groups: CON (1% DMSO vehicle control), BPF (20 mg/kg/d), BPF + CUR (20 mg/kg/d BPF + 100 mg/kg/d CUR). Data are presented as mean ± SD. * *p* < 0.05, ** *p* < 0.01 vs. CON; ^#^ *p* < 0.05, ^##^ *p* < 0.01 vs. BPF group.

Following confirmation of high-concentration BPF-induced OP, we investigated the therapeutic potential of CUR against these effects. CUR treatment significantly attenuated BPF-induced reductions in femoral BMD (Figure 5A,B). Specifically, BPF decreased femoral BMD by approximately 10.8% compared to the control group, and CUR restored approximately 43.5% of the BPF-induced loss in femoral BMD (Table S1). Histological analysis further demonstrated that CUR alleviated BPF-induced trabecular bone loss and structural deterioration (Figure 5C). Additionally, serum levels of ALP (Figure 5A) and phosphorus (Figure 6B) were also improved by CUR intervention. At the molecular level, CUR concomitantly reversed the BPF-mediated downregulation of key osteogenic markers RUNX2 and OCN, as well as p-PI3K and p-AKT (Figure 6D–H). Collectively, these findings suggest that CUR partially alleviates BPF-induced osteoporotic changes, potentially through modulation of the PI3K/AKT signaling pathway.

### 3.5. CUR Attenuates BPF-Induced Impairment of Osteogenic Differentiation In Vitro

**Figure 7:**
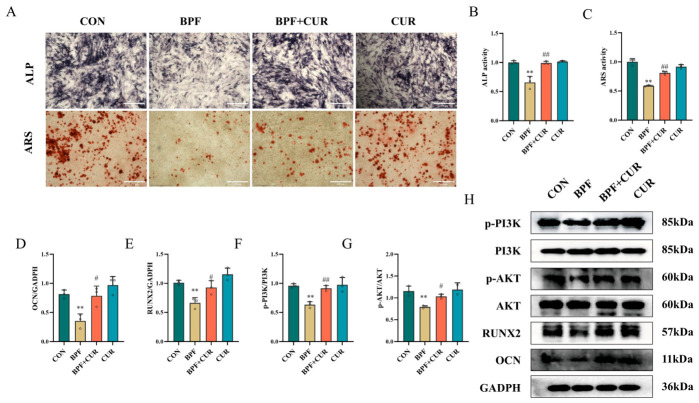
Curcumin restores BPF-impaired osteogenic function and protein expression in MC3T3-E1 cells. (**A**) Representative images showing the rescue effects of CUR on BPF-inhibited osteogenic differentiation. Top panels: ALP staining demonstrating differentiation capacity. Bottom panels: ARS staining showing mineralization potential (×40 magnification). (**B**,**C**) Quantitative analysis of ALP activity and calcium deposition. (**D**,**E**) Quantitative analysis of RUNX2 and OCN expression normalized to GAPDH. (**F**,**G**) Phosphorylation ratios of PI3K and AKT. (**H**) Representative Western blotting of p-PI3K, PI3K, p-AKT, AKT, RUNX2, OCN, and GAPDH in MC3T3-E1 cells. Treatment groups: CON (negative control), BPF (1 μM), BPF+CUR (1 μM BPF + 2 μM CUR), CUR (2 μM CUR). Data are expressed as mean ± SD (*n* = 3). ** *p* < 0.01 vs. CON; ^#^ *p* < 0.05, ^##^ *p* < 0.01 vs. BPF group.

To investigate whether BPF-induced OP and the ameliorative effects of CUR involve osteogenic differentiation, we performed in vitro experiments to evaluate the intervention effects of CUR on osteogenic differentiation and function under BPF exposure. Based on CCK-8 assays and concentrations established in previous studies, non-cytotoxic concentrations of BPF (1 μM) and CUR (2 μM) were selected for MC3T3-E1 cells (Figure S1). ALP staining revealed that BPF significantly decreased ALP activity, which was markedly restored by CUR treatment (Figure 7A,B). ARS staining demonstrated that BPF substantially attenuated mineralized nodule formation, while CUR treatment significantly enhanced mineralization (Figure 7A,C). Additionally, Western blot results showed that CUR significantly reversed BPF-induced downregulation of RUNX2, OCN, p-AKT, and p-PI3K (Figure 7D–H).

### 3.6. CUR Restores Osteogenic Differentiation via PI3K/AKT Signaling Pathway In Vitro

**Figure 8:**
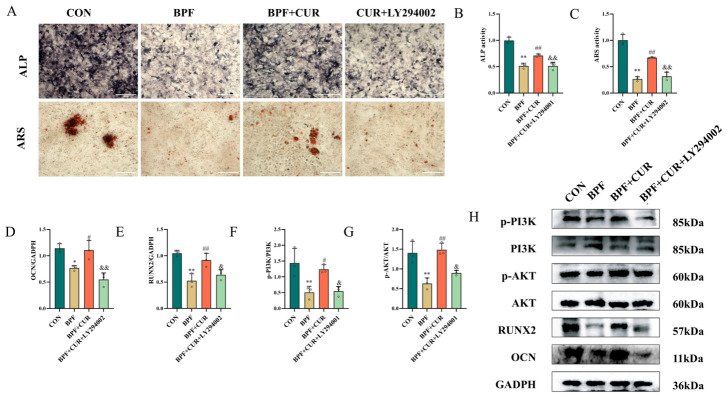
CUR restores osteogenic differentiation through PI3K/AKT signaling pathway in MC3T3-E1 cells. (**A**) Representative images showing the rescue effects of CUR on BPF-inhibited osteogenic differentiation. Top panels: ALP staining demonstrating differentiation capacity. Bottom panels: ARS staining showing mineralization potential (×40 magnification). (**B**,**C**) Quantitative analysis of ALP activity and calcium deposition. (**D**,**E**) Quantitative analysis of RUNX2 and OCN expression normalized to GAPDH. (**F**,**G**) Phosphorylation ratios of PI3K and AKT. (**H**) Representative Western blotting of p-PI3K, PI3K, p-AKT, AKT, RUNX2, OCN, and GAPDH in MC3T3-E1 cells. Treatment groups: CON (negative control), BPF (1 μM), BPF + CUR (1 μM BPF + 2 μM CUR), BPF + CUR + LY294002 (1 μM BPF + 2 μM CUR + 10 μM LY294002). Data are presented as mean ± SD (*n* = 3). * *p* < 0.05, ** *p* < 0.01 vs. CON group; ^#^ *p* < 0.05, ^##^ *p* < 0.01 vs. BPF group; ^&^ *p* < 0.05, ^&&^ *p* < 0.01 vs. BPF + CUR group.

To investigate whether CUR-mediated improvement of osteogenic differentiation involves PI3K/AKT pathway activation, we performed mechanistic studies. Prior to inhibitor experiments, we determined the non-cytotoxic concentration of LY294002 using CCK-8 assays (Figure S1). Our results demonstrated that CUR treatment effectively improved BPF-impaired ALP activity and mineralization capacity, as evidenced by ALP and ARS staining, respectively. These protective effects were abolished by co-treatment with the PI3K inhibitor LY294002 (Figure 8A–C). Western blot analysis further confirmed that CUR significantly reversed BPF-induced downregulation of osteogenic markers (RUNX2 and OCN) and PI3K/AKT signaling molecules (p-AKT and p-PI3K) (Figure 8D–H). Importantly, LY294002 treatment not only inhibited PI3K/AKT activation but also attenuated the expression of osteogenic differentiation markers (Figure 8D–H), strongly suggesting that CUR’s beneficial effects are mediated through PI3K/AKT pathway activation.

### 3.7. Validation of Key Targets by Molecular Docking of BPF and Curcumin

**Figure 9:**
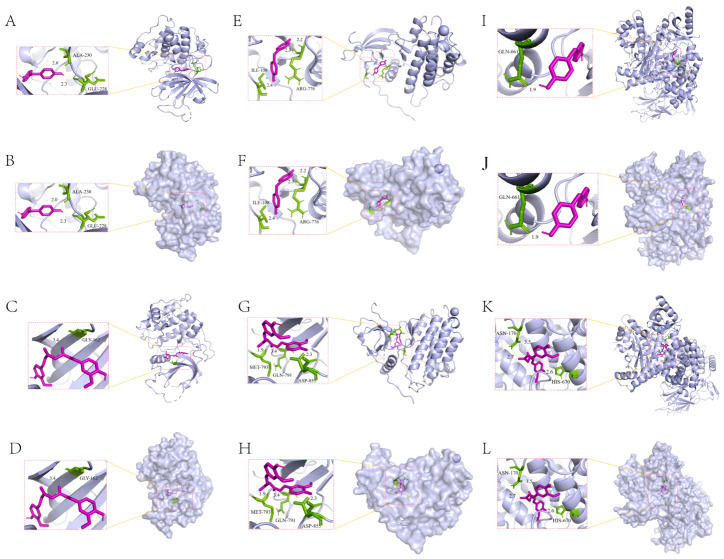
Molecular docking of BPF and curcumin with AKT1, PIK3CA, and EGFR. Structure-based molecular docking of BPF and curcumin (CUR) with key PI3K/AKT pathway proteins. Docking of BPF with AKT1 (**A**,**B**), EGFR (**E**,**F**), and PIK3CA (**I**,**J**). Docking of CUR with AKT1 (**C**,**D**), EGFR (**G**,**H**), and PIK3CA (**K**,**L**).

To further validate the interactions between active compounds and key targets, the core targets identified from network toxicology and network pharmacology analyses were intersected, revealing EGFR as a shared target. In addition, the PI3K/AKT signaling pathway was consistently identified as a significantly enriched pathway in both analyses. Based on these findings, EGFR, AKT1, and PIK3CA were selected for subsequent molecular docking with BPF and curcumin. The docking results demonstrated that both BPF and curcumin exhibited favorable binding affinities toward the three core targets (ΔG < −7 kcal/mol). Specifically, the binding free energies were as follows: BPF-AKT1, −7.239 kcal/mol (Figure 9A,B); BPF-EGFR, −7.082 kcal/mol (Figure 9E,F); BPF-PIK3CA, −7.729 kcal/mol (Figure 9I,J); CUR-AKT1, −8.530 kcal/mol (Figure 9C,D); CUR-EGFR, −7.935 kcal/mol (Figure 9G,H); CUR-PIK3CA, −8.865 kcal/mol (Figure 9K,L). These findings suggest that the two compounds have the potential to directly interact with core proteins of the PI3K/AKT signaling pathway, thereby providing structural support for the pathway identified through network analysis.

## 4. Discussion

BPA, a well-recognized endocrine-disrupting chemical, has been extensively implicated in the pathogenesis of OP [29]. However, the osteotoxic effects of its structural analogue BPF, as well as the underlying molecular mechanisms, remain insufficiently characterized [30]. Although CUR has been reported to exert anti-osteoporotic effects through multiple signaling pathways [31], its role in mitigating BPF-induced bone injury has not been systematically characterized. Therefore, this study employed a comprehensive approach integrating network toxicology, network pharmacology, and experimental validation to investigate the molecular mechanisms of BPF-induced osteoporosis and the protective effects of CUR.

Bone remodeling in humans involves continuous cellular activity, with osteoblasts playing a pivotal role in bone metabolism [32]. The migration and differentiation of osteoblast progenitor cells constitute essential physiological processes for bone formation [33]. RUNX2, a master transcription factor for skeletal development, is critically important for osteoblast differentiation during early bone formation [34]. This key regulator drives the expression of osteogenic markers including ALP, OCN, and OPN [35]. During later stages of osteogenic differentiation, highly organized matrix mineralization occurs through the coordinated expression of markers such as OPN, OCN, and bone sialoprotein (BSP) [34]. Notably, OCN not only participates in calcium metabolism but also contributes to matrix mineral deposition [34]. Therefore, RUNX2 and OCN are widely regarded as reliable indicators of osteogenic differentiation capacity. However, whether BPF induces osteoporosis through impairment of osteogenic differentiation has not been fully clarified.

In this study, network toxicology analysis identified BPF-associated targets that significantly overlapped with osteoporosis-related genes, highlighting several key nodes and enriched signaling pathways. Among these, the PI3K/AKT signaling pathway was prominently enriched, suggesting its potential central role in BPF-induced bone toxicity. Furthermore, network pharmacology analysis revealed a substantial overlap between CUR targets and osteogenesis-related pathways, with PI3K/AKT emerging as a shared critical pathway. These findings suggest that the PI3K/AKT signaling pathway may represent one of the important molecular pathways potentially involved in both BPF-induced osteotoxicity and the protective effects of CUR.

Experimental validation further substantiated these predictions. In vivo, high-dose BPF exposure significantly reduced BMD and disrupted trabecular microarchitecture in rats, accompanied by decreased serum levels of ALP, Ca, and P, indicating impaired bone formation and metabolic dysfunction. Western blot analysis demonstrated downregulation of RUNX2 and OCN in femoral tissues. Consistently, in vitro experiments using MC3T3-E1 cells showed that BPF inhibited osteoblast differentiation, as evidenced by reduced RUNX2 and OCN expression, decreased ALP activity, and diminished mineralized nodule formation. These findings are supported by previous studies showing that BPF exposure reduces bone stiffness [14] and suppresses osteogenic gene expression in human osteoblasts [35]. In addition, BPA has been shown to suppress osteoblast differentiation through estrogen receptor-mediated mechanisms [36] and to impair bone formation via oxidative stress and apoptosis induction [37]. Collectively, these observations suggest that impaired osteogenic differentiation represents a key mechanism underlying BPF-induced osteoporosis.

In the present study, BPF inhibited PI3K/AKT signaling and impaired osteogenic differentiation. Consistent with our findings, studies in other tissues have shown that bisphenol compounds can suppress PI3K/AKT pathway activity. For example, BPA has been reported to reduce PI3K and AKT phosphorylation in rat liver [38], while BPF attenuates insulin-stimulated PI3K/AKT activation in adipocytes [39]. Additionally, BPA inhibits granulosa cell proliferation and induces autophagy via PI3K/AKT pathway suppression [40]. Importantly, the effects of bisphenols on PI3K/AKT signaling are highly context-dependent. Although inhibitory effects are frequently observed in metabolic and somatic cell types, pathway activation has been reported under specific conditions, such as in immune-related tissues or at low exposure concentrations [41,42]. This variability may be attributed to dose-dependent biphasic responses, exposure duration, and cell type-specific regulatory mechanisms. Taken together, these findings suggest that bisphenol compounds may exert bidirectional regulatory effects on bone homeostasis through the PI3K/AKT pathway, by both inhibiting bone formation and promoting bone resorption. Previous studies have reported that BPA can activate AKT1 to stimulate osteoclastogenesis and enhance bone resorption [5], further supporting this dual mechanism. As a central regulatory hub in bone remodeling, dysregulation of the PI3K/AKT pathway may disrupt the balance between osteoblast and osteoclast activity, ultimately leading to impaired skeletal homeostasis and the progression of osteoporosis.

As an endocrine-disrupting chemical, BPF has been reported to exhibit multiple hormonal activities, including estrogenic, anti-estrogenic, androgenic, and anti-androgenic effects, and may interfere with endogenous hormone signaling pathways that are important for maintaining bone homeostasis [7,43]. Previous studies have suggested that disruption of hormone receptor-mediated signaling contributes to bisphenol-induced impairment of osteoblast function and skeletal abnormalities [44]. In the present study, only male rats were used, which may reduce, but does not eliminate, the influence of estrogen-dependent regulation compared with female models. Therefore, although our results support the involvement of PI3K/AKT signaling in BPF-induced osteotoxicity, the potential contribution of hormone receptor-related mechanisms cannot be completely excluded and warrants further investigation.

CUR, a bioactive polyphenol derived from turmeric, has been extensively studied for its beneficial effects on bone metabolism [23]. Previous studies have demonstrated that CUR and other dietary polyphenols can inhibit osteoclast activity or promote osteoblast function, thereby exerting positive regulatory effects on bone metabolism [45]. In addition, CUR-based nanoparticles have been reported to enhance osteogenic differentiation of bone mesenchymal stem cells under diabetic microenvironmental conditions, while simultaneously reducing trabecular bone loss and promoting bone formation [46]. Despite these promising findings, the potential protective effects of CUR against BPF-induced osteoporosis and the associated impairment of osteoblast differentiation have not yet been systematically explored.

Our findings demonstrate that CUR significantly alleviates BPF-induced osteoporotic phenotypes, including improvements in BMD, trabecular microarchitecture, and serum bone metabolism markers. Moreover, CUR reverses the BPF-induced downregulation of RUNX2 and OCN and restores osteoblast differentiation and mineralization capacity. Mechanistically, CUR partially restored the reduced phosphorylation levels of PI3K and AKT induced by BPF, which was accompanied by improved osteogenic differentiation. Notably, the protective effects of CUR were abolished by the PI3K/AKT inhibitor LY294002, further supporting the involvement of the PI3K/AKT pathway in the protective effects of CUR. Previous studies have demonstrated that pharmacological inhibition of PI3K/AKT signaling by LY294002 suppresses osteoblast proliferation, ALP activity, calcium deposition, and the expression of osteogenic markers, highlighting the essential role of this pathway in osteoblast differentiation and bone formation [47]. Consistent with our findings, previous studies have demonstrated that CUR modulates the PI3K/AKT signaling pathway across multiple tissues. For instance, in a rat model of diabetic cardiomyopathy, CUR treatment was shown to restore the reduced phosphorylation levels of PI3K and AKT to near-normal levels [48]. Similarly, in polycystic ovary syndrome (PCOS) models, CUR protects ovarian granulosa cells against hyperandrogen-induced apoptosis via reactivation of PI3K/AKT signaling [49]. Additionally, in tendon stem cells, CUR enhanced AKT phosphorylation, and its pro-differentiation effects were dependent on PI3K/AKT pathway activation [50].

This study provides integrated computational and experimental evidence that PI3K/AKT signaling is involved in BPF-induced osteogenic dysfunction and may contribute to the protective effects of CUR against BPF-induced osteoporosis.

Several limitations of this study should be acknowledged. First, while pathological observations and serum bone metabolism markers provided supportive evidence for the osteoporotic phenotype, additional skeletal evaluations, such as micro-CT analysis, TRAP staining, and trabecular biomechanical assessment, would further strengthen the characterization of bone alterations and should be considered in future studies. Second, mechanistic investigations were primarily focused on osteoblast differentiation in MC3T3-E1 cells. Since bone remodeling is regulated by the coordinated activities of osteoblasts and osteoclasts, the effects of BPF and CUR on osteoclast differentiation and bone resorption remain insufficiently characterized. In addition, osteoclast-related assessments, including RANKL/OPG measurements and TRAP staining, were not performed, warranting further investigation. Third, only male rats were used to minimize hormonal variability, and the BPF doses applied in this study (5–20 mg/kg/day in vivo, 1 μM in vitro) were higher than typical human environmental exposure levels. For example, median urinary BPF concentrations were reported as 0.35 μg/L in U.S. adults [9] and 0.08 μg/L in adults from South China [10], substantially lower than the experimental doses used here. These doses were selected based on previous studies of BPF-induced skeletal toxicity and represent commonly used approaches in mechanistic toxicology research. Therefore, caution is warranted when extrapolating these findings to real-world human exposure scenarios. Fourth, while pharmacological inhibition using LY294002 suggested involvement of the PI3K/AKT pathway in CUR-mediated protection, more rigorous validation using genetic approaches (e.g., knockdown, knockout, or overexpression) is required to establish causality. Moreover, molecular docking and network pharmacology analyses were based on Homo sapiens databases, whereas experimental validation was performed in rodent models; therefore, potential interspecies differences should be considered when interpreting the computational results. Finally, although CUR exhibited significant protective effects against BPF-induced osteoporosis, its poor oral bioavailability, rapid metabolism, and uncertain clinical dose translation may limit its therapeutic applicability.

## 5. Conclusions

Our findings demonstrate that PI3K/AKT signaling is involved in BPF-induced osteogenic dysfunction and osteoporosis. Curcumin effectively attenuates these adverse effects, potentially through restoration of PI3K/AKT pathway activity, highlighting its promise as a candidate intervention against environmentally induced bone damage.

## Data Availability

The public datasets used in this study include disease-related targets from GeneCards and OMIM, chemical and target information from PubChem, ChEMBL, CTD, SwissTargetPrediction, SEA, TargetNet, and PharmMapper, as well as gene expression data from the GEO database (accession number: GSE35958). All data sources are publicly available as described in the Methods section (Section 2.11 and Section 2.12). The experimental data generated by the authors are available from the corresponding author upon reasonable request.

## Supplementary Materials

The following supporting information can be downloaded at https://www.mdpi.com/article/10.3390/nu18142335/s1. Table S1: Bone Mineral Density Levels in Different Groups of Rats. Figure S1: Dose-dependent effects of BPF, curcumin, and PI3K inhibition on MC3T3-E1 cell viability.

## Abbreviations

The following abbreviations are used in this manuscript:

BPF	Bisphenol F
CUR	Curcumin
BMD	Bone mineral density
ALP	Alkaline phosphatase
OCN	Osteocalcin
OP	Osteoporosis
EDCs	Endocrine-disrupting chemicals
BPA	Bisphenol A
GO	Gene Ontology
KEGG	Kyoto Encyclopedia of Genes and Genomes
GSK3β	Glycogen synthase kinase-3β

## References

[1] Kanis J.A. (1994). Assessment of fracture risk and its application to screening for postmenopausal osteoporosis: Synopsis of a WHO report. WHO Study Group. Osteoporos. Int..

[2] Sözen T., Özışık L., Başaran N. (2017). An overview and management of osteoporosis. Eur. J. Rheumatol..

[3] Wang J., Yang J., Tang Z., Yu Y., Chen H., Yu Q., Zhang D., Yan C. (2023). Curculigo orchioides polysaccharide COP70-1 stimulates osteogenic differentiation of MC3T3-E1 cells by activating the BMP and Wnt signaling pathways. Int. J. Biol. Macromol..

[4] Zhivodernikov I.V., Kirichenko T.V., Markina Y.V., Postnov A.Y., Markin A.M. (2023). Molecular and Cellular Mechanisms of Osteoporosis. Int. J. Mol. Sci..

[5] Wan B., Zhou J., Teng Y., Tong Y., Zong S. (2025). Identification of a key environment-responsive gene mediating environmental impact on postmenopausal osteoporosis. Front. Public Health.

[6] Wang Z., Chen Y., Ma J., Yang Y., Li K., Jiao X., Xu B., Shi G., Wang L., Qi L. (2026). Bisphenol A induces osteoporosis by targeting LAMA4 and OLR1: Novel insights into environmental bone toxicity. Toxicology.

[7] Rochester J.R., Bolden A.L. (2015). Bisphenol S and F: A Systematic Review and Comparison of the Hormonal Activity of Bisphenol A Substitutes. Environ. Health Perspect..

[8] Liao C., Kannan K. (2014). A survey of alkylphenols, bisphenols, and triclosan in personal care products from China and the United States. Arch. Environ. Contam. Toxicol..

[9] Lehmler H.J., Liu B., Gadogbe M., Bao W. (2018). Exposure to Bisphenol A, Bisphenol F, and Bisphenol S in U.S. Adults and Children: The National Health and Nutrition Examination Survey 2013–2014. ACS Omega.

[10] Li C., Zhao Y., Chen Y., Wang F., Tse L.A., Wu X., Xiao Q., Deng Y., Li M., Kang L. (2021). The internal exposure of bisphenol analogues in South China adults and the associated health risks. Sci. Total Environ..

[11] Lu Y., Chen S., Jin H., Tang L., Xia M. (2023). Associations of bisphenol F and S, as substitutes for bisphenol A, with cardiovascular disease in American adults. J. Appl. Toxicol..

[12] Fan Y., Li S., Yang X., Bai S., Tang M., Zhang X., Lu C., Ji C., Du G., Qin Y. (2024). Multi-omics approach characterizes the role of Bisphenol F in disrupting hepatic lipid metabolism. Environ. Int..

[13] Mu X., Qi S., Liu J., Wang H., Yuan L., Qian L., Li T., Huang Y., Wang C., Guo Y. (2022). Environmental level of bisphenol F induced reproductive toxicity toward zebrafish. Sci. Total Environ..

[14] García-Recio E., Costela-Ruiz V.J., Melguizo-Rodríguez L., Ramos-Torrecillas J., Illescas-Montes R., De Luna-Bertos E., Ruiz C. (2023). Effects of bisphenol F, bisphenol S, and bisphenol AF on cultured human osteoblasts. Arch. Toxicol..

[15] Liang J., Pang L., Yang C., Long J., Liao Q., Tang P., Huang H., Wei H., Chen Q., Yang K. (2023). Effects of prenatal single and mixed bisphenol exposure on bone mineral density in preschool children: A population-based prospective cohort study. Ecotoxicol. Environ. Saf..

[16] Cauley J.A., Danielson M.E., Boudreau R.M., Forrest K.Y., Zmuda J.M., Pahor M., Tylavsky F.A., Cummings S.R., Harris T.B., Newman A.B. (2007). Inflammatory markers and incident fracture risk in older men and women: The Health Aging and Body Composition Study. J. Bone Min. Res..

[17] Zhang J., Hu W., Zou Z., Li Y., Kang F., Li J., Dong S. (2024). The role of lipid metabolism in osteoporosis: Clinical implication and cellular mechanism. Genes Dis..

[18] Song S., Guo Y., Yang Y., Fu D. (2022). Advances in pathogenesis and therapeutic strategies for osteoporosis. Pharmacol. Ther..

[19] Bai X.C., Lu D., Bai J., Zheng H., Ke Z.Y., Li X.M., Luo S.Q. (2004). Oxidative stress inhibits osteoblastic differentiation of bone cells by ERK and NF-kappaB. Biochem. Biophys. Res. Commun..

[20] Liu C., Zhang J., Ye Z., Luo J., Peng B., Wang Z. (2025). Research on the role and mechanism of the PI3K/Akt/mTOR signalling pathway in osteoporosis. Front. Endocrinol..

[21] Amalraj A., Pius A., Gopi S., Gopi S. (2017). Biological activities of curcuminoids, other biomolecules from turmeric and their derivatives—A review. J. Tradit. Complement. Med..

[22] Araújo C.C., Leon L.L. (2001). Biological activities of *Curcuma longa* L.. Mem. Inst. Oswaldo Cruz.

[23] Kotha R.R., Luthria D.L. (2019). Curcumin: Biological, Pharmaceutical, Nutraceutical, and Analytical Aspects. Molecules.

[24] Moghaddam N.S.A., Oskouie M.N., Butler A.E., Petit P.X., Barreto G.E., Sahebkar A. (2019). Hormetic effects of curcumin: What is the evidence?. J. Cell Physiol..

[25] Rongpan S., Tong-Un T., Maneesai P., Iampanichakul M., Potue P., Khamseekaew J., Prachaney P., Pakdeechote P. (2026). Luteolin attenuates vascular-renal dysfunction and fibrosis in hypertensive rats by targeting oxidative stress, inflammation, and fibrotic pathways. Biomed. Pharmacother..

[26] Jiang Q., Lei Y.H., Krishnadath D.C., Zhu B.Y., Zhou X.W. (2021). Curcumin regulates EZH2/Wnt/β-Catenin pathway in the mandible and femur of ovariectomized osteoporosis rats. Kaohsiung J. Med. Sci..

[27] Turker Yavas F., Sevil Kilimci F., Akkoc A.N., Sahiner H.S., Bardakci Yilmaz Ö. (2024). Melatonin’s protective role against Bisphenol F and S-induced skeletal damage: A morphometric and histological study in rat. Ann. Anat..

[28] Chen Z., Xue J., Shen T., Mu S., Fu Q. (2016). Curcumin alleviates glucocorticoid-induced osteoporosis through the regulation of the Wnt signaling pathway. Int. J. Mol. Med..

[29] Chin K.Y., Pang K.L., Mark-Lee W.F. (2018). A Review on the Effects of Bisphenol A and Its Derivatives on Skeletal Health. Int. J. Med. Sci..

[30] Park S.Y., Kong S.H., Kim K.J., Ahn S.H., Hong N., Ha J., Lee S., Choi H.S., Baek K.H., Kim J.E. (2024). Effects of Endocrine-Disrupting Chemicals on Bone Health. Endocrinol. Metab..

[31] Wang K. (2024). The potential therapeutic role of curcumin in osteoporosis treatment: Based on multiple signaling pathways. Front. Pharmacol..

[32] Yao D., Huang L., Ke J., Zhang M., Xiao Q., Zhu X. (2020). Bone metabolism regulation: Implications for the treatment of bone diseases. Biomed. Pharmacother..

[33] Salhotra A., Shah H.N., Levi B., Longaker M.T. (2020). Mechanisms of bone development and repair. Nat. Rev. Mol. Cell Biol..

[34] Amarasekara D.S., Kim S., Rho J. (2021). Regulation of Osteoblast Differentiation by Cytokine Networks. Int. J. Mol. Sci..

[35] García-Recio E., Costela-Ruiz V.J., Illescas-Montes R., Melguizo-Rodríguez L., García-Martínez O., Ruiz C., De Luna-Bertos E. (2023). Modulation of Osteogenic Gene Expression by Human Osteoblasts Cultured in the Presence of Bisphenols BPF, BPS, or BPAF. Int. J. Mol. Sci..

[36] Thent Z.C., Froemming G.R.A., Muid S. (2018). Bisphenol A exposure disturbs the bone metabolism: An evolving interest towards an old culprit. Life Sci..

[37] Shi X., Wu K., Liu C., Cao K., Zhang Q., Wu W., Luo C., Huang W. (2024). Preliminary investigation into the impact of BPA on osteoblast activity and bone development: In vitro and in vivo models. Environ. Pollut..

[38] Mohsenzadeh M.S., Razavi B.M., Imenshahidi M., Tabatabaee Yazdi S.A., Mohajeri S.A., Hosseinzadeh H. (2021). Potential role of green tea extract and epigallocatechin gallate in preventing bisphenol A-induced metabolic disorders in rats: Biochemical and molecular evidence. Phytomedicine.

[39] Chen H., Li J., Zhang Y., Zhang W., Li X., Tang H., Liu Y., Li T., He H., Du B. (2022). Bisphenol F suppresses insulin-stimulated glucose metabolism in adipocytes by inhibiting IRS-1/PI3K/AKT pathway. Ecotoxicol. Environ. Saf..

[40] Li C., Cui Z., Liu Z., Fan H., Lan Y., Luo J., Ruan F., Huang Y., Chu K., Wu Y. (2024). MiR-204 regulates autophagy and cell viability by targeting BDNF and inhibiting the NTRK2-dependent PI3K/Akt/mTOR pathway in a human granulosa cell line exposed to bisphenol A. Ecotoxicol. Environ. Saf..

[41] Dong Y., Gao L., Sun Q., Jia L., Liu D. (2023). Increased levels of IL-17 and autoantibodies following Bisphenol A exposure were associated with activation of PI3K/AKT/mTOR pathway and abnormal autophagy in MRL/lpr mice. Ecotoxicol. Environ. Saf..

[42] Liu J., Wang H., Hou X., Fan L., Yang F., Dai Y., Deng Y., Fu Z., Shu X., Sun B. (2023). Bisphenol P and bisphenol M promote triple-negative breast cancer metastasis through activation of AKT pathways. Sci. Total Environ..

[43] Turan S. (2021). Endocrine disrupting chemicals and bone. Best Pract. Res. Clin. Endocrinol. Metab..

[44] Miki Y., Hata S., Nagasaki S., Suzuki T., Ito K., Kumamoto H., Sasano H. (2016). Steroid and xenobiotic receptor-mediated effects of bisphenol A on human osteoblasts. Life Sci..

[45] Kunihiro A.G., Luis P.B., Frye J.B., Chew W., Chow H.H., Schneider C., Funk J.L. (2020). Bone-Specific Metabolism of Dietary Polyphenols in Resorptive Bone Diseases. Mol. Nutr. Food Res..

[46] Li Y., Cai Z., Ma W., Bai L., Luo E., Lin Y. (2024). A DNA tetrahedron-based ferroptosis-suppressing nanoparticle: Superior delivery of curcumin and alleviation of diabetic osteoporosis. Bone Res..

[47] Zheng Z., He Y., Long L., Gan S., Chen S., Zhang M., Xu J., Fu R., Liao Y., Zhu Z. (2022). Involvement of PI3K/Akt signaling pathway in promoting osteogenesis on titanium implant surfaces modified with novel non-thermal atmospheric plasma. Front. Bioeng. Biotechnol..

[48] Ren B.C., Zhang Y.F., Liu S.S., Cheng X.J., Yang X., Cui X.G., Zhao X.R., Zhao H., Hao M.F., Li M.D. (2020). Curcumin alleviates oxidative stress and inhibits apoptosis in diabetic cardiomyopathy via Sirt1-Foxo1 and PI3K-Akt signalling pathways. J. Cell Mol. Med..

[49] Zhang Y., Wang L., Weng Y., Wang D., Wang R., Wang H., Wang L., Shen S., Wang H., Li Y. (2022). Curcumin Inhibits Hyperandrogen-Induced IRE1α-XBP1 Pathway Activation by Activating the PI3K/AKT Signaling in Ovarian Granulosa Cells of PCOS Model Rats. Oxid. Med. Cell Longev..

[50] Zhang Z., Zhang Y., Wang H., Li B., Cao R., Li Y., Cui S., Zhang W. (2024). Curcumin Improves Functional Recovery of Ruptured Tendon by Promoting Tenogenesis via PI3K/Akt Signaling. Stem Cells Transl. Med..

