# Supplementary material for: Curcumin Attenuates Bisphenol F-Induced Osteoporosis and Osteogenic Dysfunction via PI3K/AKT Pathway Activation
Source: Nutrients. 2026 Jul 16;18(14):2335. doi: 10.3390/nu18142335 (PMC13416212; doi:10.3390/nu18142335)
Supplement: Supplementary file 1 [file nutrients-18-02335-s001.zip › nutrients-4343076-supplementary.pdf]

Curcumin Attenuates Bisphenol F-Induced Osteoporosis and Osteogenic Dysfunction via PI3K/AKT Pathway Activation

Supplementary Online Content

Supplementary Results

Table S1. Bone Mineral Density Levels in Different Groups of Rats

Figure S1. Dose-dependent effects of BPF, curcumin, and PI3K inhibition on MC3T3-E1 cell viability.

Table S1. Bone Mineral Density Levels in Different Groups of Rats

Group	Femoral Bone Mineral Density (kg/m^2)
CON	0.213±0.004
BPF	0.190±0.010**
BPF+CUR	0.200±0.010$^{\#\#}$

Note: Data are presented as mean ± SD (n = 6). **P < 0.01 versus the CON group; $^{\#\#}$P < 0.01 versus the BPF group.

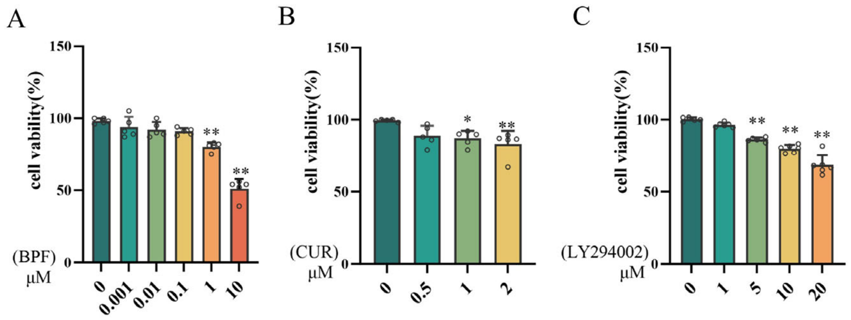

Figure S1. Dose-dependent effects of BPF, curcumin, and PI3K inhibition on MC3T3-E1 cell viability. Effects of BPF, CUR, and LY294002 on MC3T3-E1 cells viability. (A) Cell viability after 48 h treatment with varying BPF concentrations. (B) Cell viability after 48 h treatment with varying CUR concentrations. (C) Cell viability after 48 h treatment with different concentrations of LY294002 (PI3K inhibitor). Data are expressed as mean ± SD (n = 5). $^{*}P < 0.05$, $^{**}P < 0.01$ vs CON.
